# Novel Mode of Defective Neural Tube Closure in the Non-Obese Diabetic (NOD) Mouse Strain

**DOI:** 10.1038/srep16917

**Published:** 2015-11-23

**Authors:** J. Michael Salbaum, Claudia Kruger, Jacalyn MacGowan, Nils J. Herion, David Burk, Claudia Kappen

**Affiliations:** 1 Pennington Biomedical Research Center, Department of Regulation of Gene Expression, 6400 Perkins Road, Baton Rouge, LA 70808, USA; 2 Pennington Biomedical Research Center Department of Developmental Biology, 6400 Perkins Road, Baton Rouge, LA 70808, USA; 3 Pennington Biomedical Research Center Cell Biology and Bioimaging Core Facility, 6400 Perkins Road, Baton Rouge, LA 70808, USA

## Abstract

Failure to close the neural tube results in birth defects, with severity ranging from spina bifida to lethal anencephaly. Few genetic risk factors for neural tube defects are known in humans, highlighting the critical role of environmental risk factors, such as maternal diabetes. Yet, it is not well understood how altered maternal metabolism interferes with embryonic development, and with neurulation in particular. We present evidence from two independent mouse models of diabetic pregnancy that identifies impaired migration of nascent mesodermal cells in the primitive streak as the morphogenetic basis underlying the pathogenesis of neural tube defects. We conclude that perturbed gastrulation not only explains the neurulation defects, but also provides a unifying etiology for the broad spectrum of congenital malformations in diabetic pregnancies.

Failure to close the neural tube results in birth defects^1^,^2^, with severity ranging from asymptomatic spina bifida occulta and surgically correctable cases of spina bifida to lethal conditions like exencephaly and anencephaly. Approximately 400 genes have been identified in the mouse where mutations either cause or contribute to neurulation defects^3^,^4^. In contrast, comparatively few genetic risk factors are known in humans^5^, highlighting the critical role of environmental risk factors, such as folic acid deficiency^6^ or maternal diabetes^7^,^8^,^9^,^10^. However, despite improved dietary folic acid supply and better glycemic control, the incidence of neural tube defects (NTDs) has been reduced only partially^11^,^12^. The extant risk for neural tube defects demands a better understanding how environmental factors interfere with embryonic development in general, and with neurulation in particular.

The Non-Obese Diabetic (NOD) strain of mice, which is prone to spontaneously develop autoimmune diabetes, is an established model for human type I diabetes^13^,^14^. Embryos of diabetic NOD pregnancies are afflicted with a very high rate of neural tube defects^15^ (NTDs), and heart defects^16^,^17^, another hallmark of diabetes teratogenicity. We here report that periconceptional supplementation with folinic acid in NOD diabetic dams reduced NTD incidence from 40.2% to 21% (p = 0.006, Supplemental Fig. 1). Thus, in parallel to human pregnancies, NTDs in this model are partially folate-responsive.

Unexpectedly, we found that in embryos from hyperglycemic NOD dams, the neural plate displayed protruding ectopic tissue in various locations along the anterior-posterior axis (Fig. 1a–f). Protrusions were strictly limited to diabetic pregnancies, and occurred in 25.3% of embryos at 8.5 days of gestation (E8.5). To test whether protrusions were unique to the NOD strain, we induced hyperglycemia in females of the FVB strain with Streptozotocin^18^, resulting in an NTD incidence of 21.6%^19^. At E8.5, 12.9% of hyperglycemia-exposed FVB embryos displayed protrusions (Fig. 1g,h). The overall appearance, location along the anterior-posterior axis, and internal organization (Fig. 1k) of protrusions were strikingly similar to the phenotype of exposed NOD embryos. Thus, these malformations are not a peculiarity of the genetic background of the NOD strain, but arise from the severe maternal hyperglycemia (Supplemental Table 1) common to both experimental models.

Imaging by 2-photon confocal microscopy, three-dimensional reconstruction of whole embryos from optical sections (Fig. 1i), and subsequent generation of single-plane views, allowed closer examination of the juncture between a protrusion and the embryo (Fig. 1j,k). We detected contiguity between the protrusion and the neural plate, with the outer layer resembling neuroepithelium. The core of a protrusion displayed lower cell density, reminiscent of mesenchymal character. This suggested that protrusions are not exclusively composed of neuroepithelial cells.

To determine the origin of protrusions, we performed gene expression profiling on microdissected protrusion tissue from the FVB model, using 3’-expression tag sequencing. For comparison, we laser-microdissected open neural tube immediately anterior of closure site 1 (Supplemental Fig. 2a–c). Analysis of *Noto* gene expression indicated that neural tube samples were free of potentially contaminating notochord (Supplemental Fig. 2d–h). We identified 799 genes with statistically significant differential expression in protrusions compared to open neural tube (Fig. 2a, Supplemental Figure 2j), and confirmed the sequencing-based observations by quantitative RT-PCR for selected genes (Fig. 2b). Hierarchical clustering demonstrated a clear distinction of expression profiles between protrusions and open neural tube (Supplemental Fig. 2i). Annotations for the 570 genes with predominant expression in protrusions were significantly enriched for the GO terms “mesoderm formation” and “mesoderm development”.

For 85 of these genes, expression patterns at Theiler stages 11 to 13 (which correspond to gestational days E7.5 and E8.5) have been reported previously (MGI, http://www.informatics.jax.org/): 22 genes are known to be expressed in neuroectoderm and mesoderm, 33 are limited to mesoderm, 10 are expressed in the node, and 28 genes in the primitive streak (Supplementary Table 4). These results demonstrate that protrusions contain mesoderm, and they link protrusions to molecular networks that are active during gastrulation: Protrusions featured expression of genes known to be involved in early gastrulation, such as *Nodal* and *Furin*, which is required for Nodal activation^20^, and of components involved in maintenance of the primitive streak^21^, such as *Fgf8, Wnt3a, and T/brachyury*. We also detected targets of *T*, e.g. *Cdx2*, *Axin2*, and *Lef1*, together with 57 genes in the regulatory networks driven by *T *22,23, as well as 153 downstream targets of *Cdx2*^22^ (Supplemental Table 5); these included known axial, paraxial, and lateral mesoderm markers. Presence of these regulatory networks indicates that the mesenchymal cells in the protrusions have undergone the entire currently known mesoderm specification program.

Analyses of NOD embryos by *in situ* hybridization at E8.5 (Fig. 3) revealed parallels between protrusions arising from spontaneously initiating and chemically induced maternal hyperglycemia, respectively. *Sox2*, a marker for the epiblast/neuronal lineage^24^, was present throughout the outer layer of the protrusion, whereas *T*, a marker for primitive streak and nascent mesoderm^25^, was extended into the proximal core of the protrusion. *Tbx6*, a marker for committed mesoderm^24^,^26^, was found in the primitive streak and migrating cells of the mesodermal wings. *Tbx6* expression in the core of a protrusion was reminiscent of proper mesoderm development^27^: expression was strongest where *T* expression had already been extinguished. Overall, these data indicate that migration of mesodermal cells is not completely blocked, but impaired locally around the protrusion.

The ectopic mesoderm in the protrusions could result from altered proliferation of newly generated mesodermal cells, or from disoriented migration of nascent mesoderm. Histological analyses support the second possibility: we did not find evidence for increased cell proliferation, as staining for the mitosis marker Phospho-Histone 3 did not reveal enrichment in protrusions compared to the rest of the embryo. Instead, we detected deposition of Laminin between mesodermal cells within and at the base of a protrusion (Fig. 4). This is paralleled by protrusion-prevalent expression of *Laminin* α*5*, *Nidogen 2* (components of the basal lamina^28^,^29^), and *Integrin* α*6*, a receptor for Lama5^30^, and is consistent with a previous report of elevated expression of extracellular matrix components in rat embryos exposed to hyperglycemia conditions^31^. These data implicate altered cell adhesion or impaired migration of mesodermal cells in the formation of protrusions.

Evidence for impaired migration came from explant cultures (Fig. 5) under conditions that support migration and differentiation of mesoderm, confirmed by virtue of staining for Vimentin^32^ (Fig. 5b,c). In these cultures, outgrowth from posterior tissue explants of diabetes-exposed NOD embryos at E7.5, or at E8.5, was significantly reduced compared to migration from explants of embryos from normoglycemic NOD pregnancies (Fig. 6a). Furthermore, for diabetes-exposed embryos, we compared cultures of dissected protrusions to explants of the adjacent posterior tissue at E8.5. Cells from protrusions either failed to migrate away from the explant, or migrated significantly less compared to the outgrowth observed from the corresponding posterior tissue explant (compare within green frames: Fig. 5g,h,i to j,k,l, and Fig. 5m,n,o to p,q,r, respectively; and Fig. 6b). The reduced migration of cells away from protrusions cannot be attributed to developmental immaturity, as cells from earlier embryos exhibit comparable migratory capacity in these assays (Supplemental Fig. 3a); the extent of outgrowth was also uncorrelated to size of the starting explant (Supplemental Fig. 3b). We therefore conclude that the exposure to maternal diabetes is responsible for the impaired migratory capacity of mesoderm in protrusions. Finally, outgrowth of explants from diabetes-exposed NOD E8.5 embryos was reduced in comparison to explants of diabetes-exposed E8.5 embryos of the FVB strain (Fig. 6c), which could explain the higher incidence of protrusions in the NOD model compared to FVB.

These results also demonstrate that the culture conditions, including supportive extracellular matrix and growth factors present in fetal calf serum, are not sufficient to rescue the cell migration deficiencies during the 26 hours of culture of the explants. Culture at lower glucose concentrations had no effect on outcome (p = 0.23 for E7.5, p = 0.22 for E8.5). Thus, the explant cultures confirm our conclusion that the exposure in utero to maternal diabetes causes impaired cell migration and reduces egress from the primitive streak, which in the most severely affected individuals creates protrusions from the neural plate.

Protrusions have also been reported in 20 genetic models involving loss-of-function for the *Cripto* (*Tdgf1*), *Eomes*, *Fgf8*, *Fgfr1*, *Lrp5*/*Lrp6*, *Map4k4* (*Nik*), *Mesp1*/*Mesp2*, *Mixl1*, *Pten*, *Rac1*, *Ship2*, *T*, *Talin*, and *Tcf3* genes^33^,^34^,^35^,^36^,^37^,^38^,^39^,^40^,^41^,^42^,^43^,^44^,^45^,^46^,^47^,^48^,^49^, or ENU-induced mutations in the *Axin2*, *Nckap1* (*Nap1*), *Supt20* (*p38IP*) and *Udgh* genes^50^,^51^,^52^,^53^ (reviewed in^54^). Impaired mesoderm migration was implicated as mechanistic basis for such cell accumulations, and epiblast-specific ablation of Pten^43^ and Rac1^45^ identified those genes as cell-autonomous regulators of mesoderm migration. Among 15 protrusion mutants where embryos survive to later stages of development, common outcomes are open neural tube or spina bifida (6 models), defective heart development (9 models), and caudal growth defects (9 models)^54^. In contrast to loss-of-function models in which expression is abolished, we detected expression of all these genes in diabetes-exposed embryos, with *Axin2*, *Fgf8*, *Mesp1*, *Mesp2*, *Mixl1*, and *T* particularly prevalent in protrusions (Fig. 2a and Supplemental Table 2), indicating that defective migration in our exposure models is not due to loss of mesodermal gene expression.

Intriguingly, protrusions formed at discrete anterior-posterior locations rather than along the entire primitive streak. Consistent with prior data^55^, we found that two thirds of NTDs in NOD embryos (~26% of all embryos) involve the trunk region of the embryo; this rate is comparable to a protrusion incidence of 25%, of which almost all appear in the territory covered by the primitive streak. In the FVB model, half the NTDs (~11% of all embryos) involve the trunk (unpublished observations), a rate that again parallels the protrusion incidence (12.9%). Thus, in both diabetes models, defective mesoderm migration can account for the vast majority of trunk and caudal neural tube defects in mouse diabetic pregnancies. Protrusions in more anterior locations were detected only occasionally (e.g. Fig. 1F and insert). Even within the primitive streak territory, protrusions were limited to unique locations, possibly indicative of a limited time window for perturbations that contribute to the formation of protrusions.

There are three possibilities how protrusions can cause neural tube defects: (i) by preventing formation of the medial hinge point that is required for initial bending of the neural plate^56^,^57^, (ii) by compromising elevation and bending of future neuroepithelium^57^ due to diminished cell migration into the underlying mesoderm, and (iii) by physically interfering with the closure of the neural tube at the dorsal midline. Given that these alternatives are not mutually exclusive, they require further investigation. Close examinations and histological analyses of embryos with protrusions (Fig. 7) revealed properly closed neural tubes rostral to the protrusion, with neurulation failure caudal to the protrusion, indicating that in these cases the protrusions physically interfered with closure of the neural tube.

In this work, we have identified impaired mesoderm migration as the morphogenetic failure underlying the pathogenesis of NTDs. Since these NTDs are caused by an environmental risk factor, maternal metabolic disease, our findings imply that mesoderm migration is sensitive to metabolic state. Mesoderm migration also appears to be responsive to composition of the maternal diet, as we previously demonstrated that diet modulates the rate of NTDs in the FVB model^19^. In the NOD strain, NTD incidence is reduced by supplementation of folinic acid, as shown above, similar to the beneficial effects of folic acid in STZ-induced diabetic mouse pregnancies^58^. These findings support the conclusion that metabolic factors can affect mesoderm formation and migration, and -together with the results from our molecular analyses- identify novel cellular and molecular targets for the prevention of neural tube and other birth defects.

The most characteristic congenital malformations in human diabetic pregnancies are heart defects, neural tube defects, and caudal growth defects^7^,^8^,^9^,^10^, and have been postulated to arise before the 7th week of pregnancy^7^. Our results support the proposition that perturbed mesoderm migration during gastrulation is the common etiology for these seemingly heterogeneous birth defects^59^,^60^: (i) neural tube defects arise as a consequence of impaired mesoderm migration, as demonstrated here; (ii) early heart progenitors originate and migrate from the primitive streak^61^,^62^; and (iii) caudal growth defects are also consistent with altered mesoderm formation and migration^63^,^64^ in the posterior primitive streak. Similarly, mesodermal deficiencies are believed to underlie the vertebral, cardiac, renal and limb malformations of VACTERL^65^ and axial mesodermal dysplasia^66^ phenotypes, which have been linked to maternal diabetes^67^,^68^,^69^,^70^. Thus, our discovery of aberrant mesoderm migration during gastrulation in two different mouse models of Type I diabetes provides a unifying cellular mechanism that can explain both the developmental timing and the morphogenetic origin of the most common structural anomalies in diabetic embryopathy.

## Methods Summary

All animal experiments were performed with prior approval of the Pennington Biomedical Research Center IACUC and in accordance with the “Guide for the care and use of laboratory animals” of the United States National Institutes of Health. Embryos were prepared at 8.5 days of gestation for histological or molecular analysis from hyperglycemic NOD or FVB dams, as well as from strain-matched normoglycemic control dams. Embryos with malformations were fixed, stained with DAPI, and imaged by 2-photon confocal microscopy. Optical sections were used to generate three-dimensional reconstructions of individual embryos using Imaris software. To determine etiology and identity of protrusion tissue, we performed gene expression profiling by expression tag sequencing on an AB SOLiD 5500XL sequencer; expression profiles were compared between protrusion tissue and open neural plate prepared by laser microdissection immediately anterior of neural tube closure site 1. Sequence reads were mapped (RefSeq RNA, mm9) using SOLiDSAGE to generate count data for each gene. Differential gene expression was determined using DESeq, with validation of select genes by qPCR. *In situ* hybridizations and immunohistochemical analyses were performed on cryosections following established protocols. Migratory capacity of cells in protrusions and posterior embryonic tissue was assessed in explant culture, using time-lapse video and phase contrast microscopy.

## Additional Information

**Accession codes:** The SAGE results have been deposited in the Gene Expression Omnibus database under accession number GSE53075.

**How to cite this article**: Salbaum, J. M. *et al.* Novel Mode of Defective Neural Tube Closure in the Non-Obese Diabetic (NOD) Mouse Strain. *Sci. Rep.* **5**, 16917; doi: 10.1038/srep16917 (2015).

## Supplementary Material

Supplementary Movie S1

Supplementary Information

## Figures and Tables

**Figure 1:**
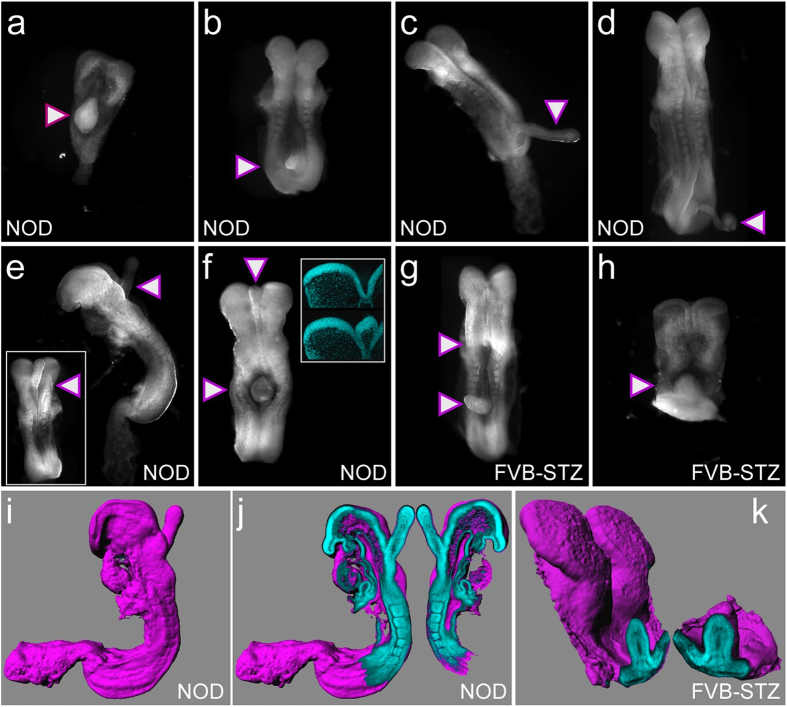
Neural plate protrusion phenotype in embryos from diabetic pregnancies. (**a**) through (**d**), Embryos from diabetic pregnancies of the NOD strain with protrusions (triangles), generally in caudal locations. (**e**), NOD embryo with a protrusion at the hindbrain level, rostral to the neural tube closure front; lateral view; insert: view of the dorsal surface of the embryo. (**f**), NOD embryo with two protrusions: a small one at the mid/forebrain region, and a larger one in the trunk area. Insert: virtual sections through unaffected (top) and affected (bottom) neural plate areas. Compared to the unaffected region, the area affected by a protrusion displays an overall normal organization with the exception of a bulge at the midline. This bulge has an outer layer similar to and contiguous with the neuroepithelium, and a core where cell nuclei are sparse. (**g**,**h**), Embryos from diabetic pregnancies of the FVB strain display protrusions similar to those observed in the NOD strain. The embryo in panel g has two such malformations: a small one at the caudal end of the prospective hindbrain, and a larger one in the caudal trunk region. (**i–k**), Images derived from 3-dimensional reconstruction of confocal imaging data of whole embryos stained with DAPI. (**i**), Same embryo as in panel e, shown with a surface (purple) as calculated from volume data. The protrusion at the hindbrain level is clearly discernible. (**j)**, Two aspects of a virtual parasagittal plane of section in ‘open-book’ presentation. The protrusion is contiguous with the neuroepithelium, with the core exhibiting a lower density of DAPI-stained cell nuclei, reminiscent of mesenchymal character. (**k**), ‘Open-book’ presentation of the FVB embryo shown in panel h. The morphological appearance of protrusions is similar in diabetes-exposed NOD and FVB embryos.

**Figure 2:**
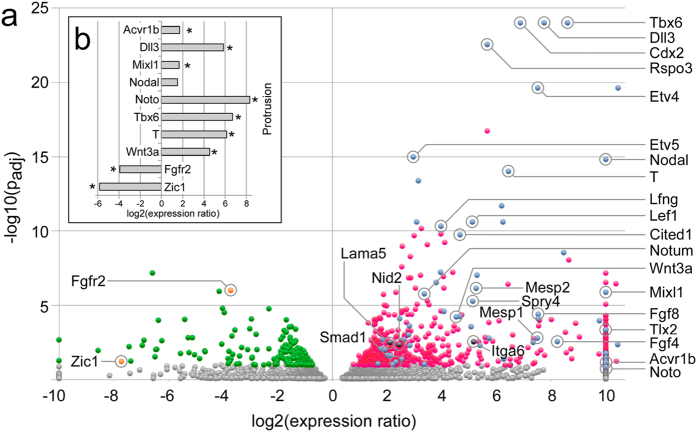
Molecular analysis of neural plate protrusions. (**a**) Transcriptome profiling was performed by 3’-expression tag sequencing (SAGE) on an AB SOLiD 5500XL next generation sequencer. Comparisons between 3 individual protrusions (n = 3) and open neural tube neuroepithelium from 4 embryos (n = 4) were done using DESeq. Data are shown as a volcano plot, with statistical significance expressed as –log10(padj) plotted against the expression ratio (protrusion vs. neural tube). Gray color represents genes that did not reach statistical significance (Benjamini-Hochberg correction); red labels genes with expression prevalence in the protrusions; among these genes, blue color indicates genes with a known role in mesoderm development. Green indicates genes with prevalence in the open neural tube, with orange color indicating two genes of interest. Genes where expression was completely absent on one side of the paradigm were plotted at a log2 of either 10 or −10. (**b)** Validation of the expression difference of selected genes by quantitative real-time PCR. Eight genes with prevalence in protrusions (*Acvr1b*, *Dll3*, *Mixl1*, *Nodal*, *Noto*, *T*, *Tbx6*, and *Wnt3a*) were chosen for validation, and two genes (*Fgfr2*, *Zic1*) with expression predominant in the open neural tube. The same samples were analyzed from which the sequencing data were derived; thus, we measured gene expression levels in four technical replicates each of three protrusions, and in four technical replicates each of 4 neuroepithelium samples (from 4 individual embryos). Bars represent expression ratio between protrusions and neural tube expressed as log2. Statistical significance in a t-test is indicated by a star symbol. Expression ratios obtained from the sequencing experiment were confirmed for all candidate genes, except for *Nodal*, which exhibited the expected direction for the expression difference, but did not reach statistical significance.

**Figure 3:**
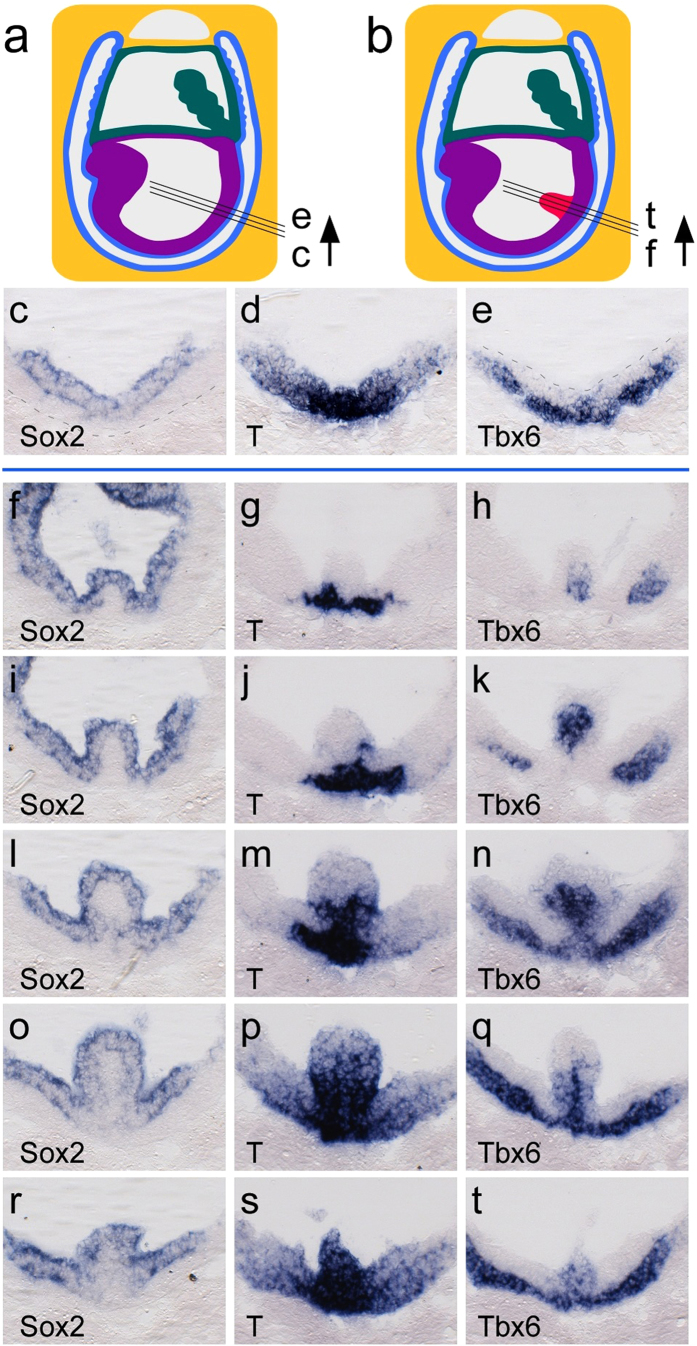
Histological analyses of neural plate protrusions. (**a**) Schematic of a mouse embryo at Theiler stage 11 (http://www.emouseatlas.org/). Black lines indicate the plane of sectioning for sections shown in panels (**c**–**e**). Colors: purple – embryonic ectoderm and mesoderm; teal – amnion and allantois; blue – endoderm and yolk sac; yellow – deciduum. (**b**) Representation of a mouse embryo with a protrusion (red); black lines indicate series of sectioning planes for sections shown in panels (**f**-**t)** in rostral to caudal sequence, through the protrusion. (**c**) Section through the posterior region of an embryo from a normoglycemic NOD pregnancy at Theiler stage 11 after *in situ* hybridization with a probe for *Sox2*. The *Sox2* signal is restricted to the epiblast/ectodermal layer. Stippled line represents the boundary of the embryo. (**d**) Adjacent section stained with a probe for *T*, revealing *T* expression in primitive streak and migrating nascent mesoderm. (**e**) Adjacent section developed with a probe for Tbx6, showing *Tbx6* expression in the mesendodermal layer in the primitive streak, and the mesodermal wings. (**f**-**t**) Series of adjacent sections through an embryo from a diabetic NOD pregnancy at Theiler stage 11 featuring a protrusion. Panel (**f**) shows the initial rostral aspect of the protrusion, whereas panel (**t**) displays the most caudal section in the sequence. (**f**,**i**,**l**,**o**,**r)**, Sections probed for *Sox2* expression, which is detected in the outer layer of the protrusion. (**g**,**j**,**m**,**p**,**s**), Adjacent sections from the same embryo revealing expression of *T*. At the rostral aspect, *T* is detected at the base of the protrusion in a region reminiscent of the primitive streak. In the center of the protrusion (panel (**m)**), *T* is expressed in the outer layer as well as in the core. (**h**,**k**,**n**,**q**,**t**). Adjacent sections probed for expression of *Tbx6*. *Tbx6* is prominently detected in the core of the protrusion, and is absent from the outer layer. Presence of *Tbx6* in the mesodermal wings represents the normal expression for this gene in domains where the expression of *T* has already ceased.

**Figure 4:**
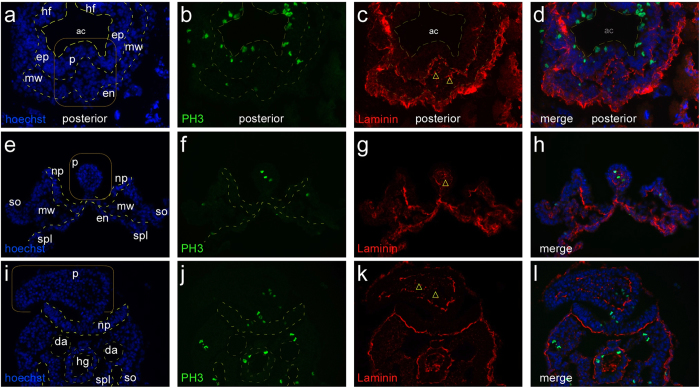
Protrusions feature internal deposition of laminin. Embryos at three different stages of development, featuring protrusions: E8.0, headfold stage (**a**–**d**) E8.5 with open neural plate in the caudal region (**e**–**h**) and E9.5 with a large protrusion interfering with neural tube closure (**i**–**l**). Sections were stained for DNA ((**a**,**e**,**i**); blue, Hoechst 33342), phosphorylated Histone 3 ((**b**,**f**,**j**); PH3, green), and Laminin ((**c**,**g**,**k**); red); merged color images are shown in (**d**,**h**,**l**) respectively. Abbreviations: ac, amniotic cavity; da, dorsal aorta; en, endoderm; ep, epiblast layer; hf, headfold; hg, hindgut; mw, mesodermal wing; np, neural plate; p, protrusion; so, somatopleura; spl, splanchnopleura. All protrusions, from the early stage to the mature stage, feature internal accumulations of laminin in the mesenchyme (yellow triangles). Furthermore, the outer cell layer is typically separated from the internal mesenchyme by a laminin-positive layer of extracellular matrix.

**Figure 5:**
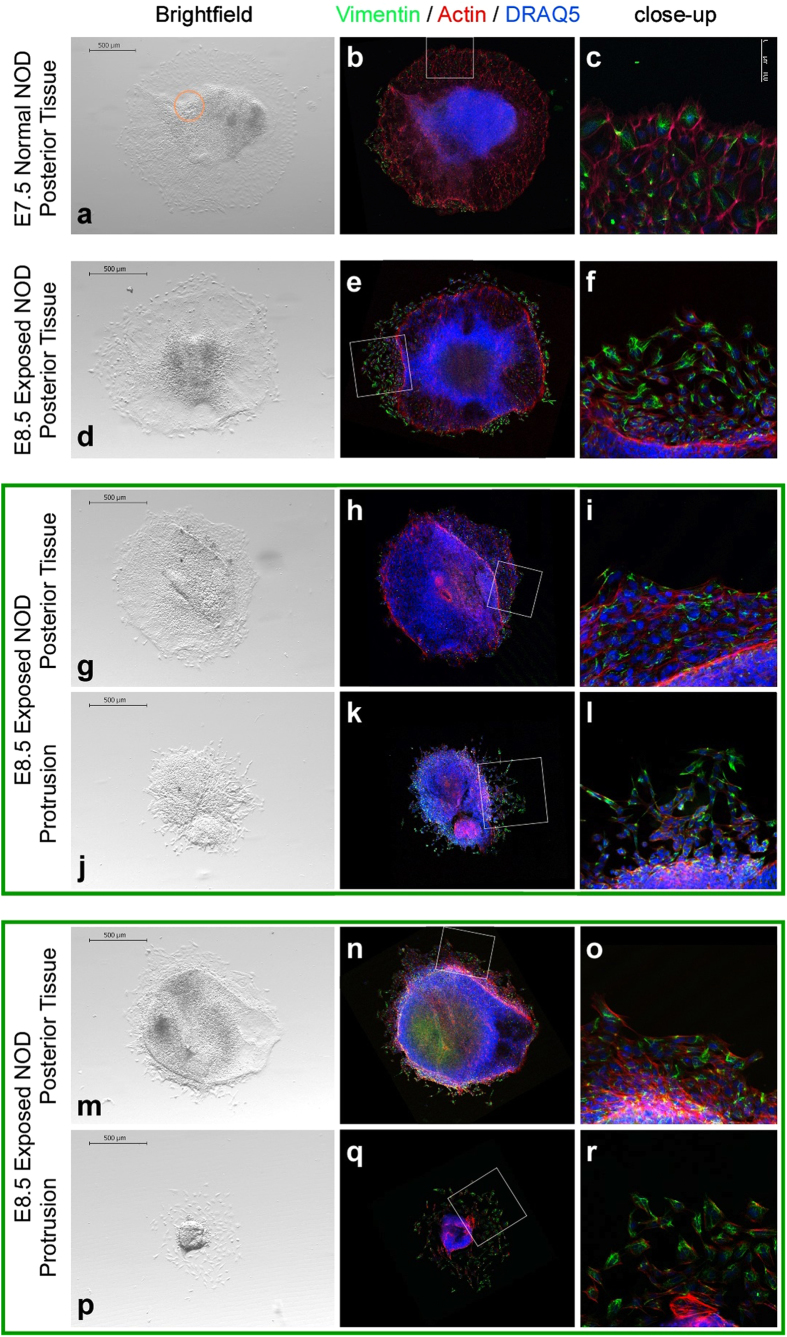
Explant cultures of protrusions and posterior embryonic tissue. Images of explant cultures. Fixed explants were stained with an antibody against Vimentin (green), Phalloidin (red) to detect Actin, and DRAQ5 (blue) to localize cell nuclei. Culture conditions supported migration and differentiation of mesoderm, including cardiac mesoderm, as evidenced by formation of rhythmically contractile centers in primitive streak explants of E7.5 embryos from normal NOD pregnancies (time-lapse video in Supplemental Information). Insets identify the area depicted in the close-up images. (**a**) The explant from a normal E7.5 NOD embryo had an area of rhythmically contracting myocytes (orange circle). (**b**) The same explant after staining, in darkfield contrast. (**c**) Close-up magnification of inset area. (**d**–**f**) Explant from a diabetes-exposed E8.5 NOD embryo. (**g**–**l**,**m**–**r**) Corresponding explants of posterior tissue and protrusion from the same embryo are grouped together by a green frame. Compared to posterior tissue from the same embryo, respectively, (**g**–**i**,**m**–**o**) protrusions exhibit very little outgrowth (**j**–**l**,**p**–**r**).

**Figure 6:**
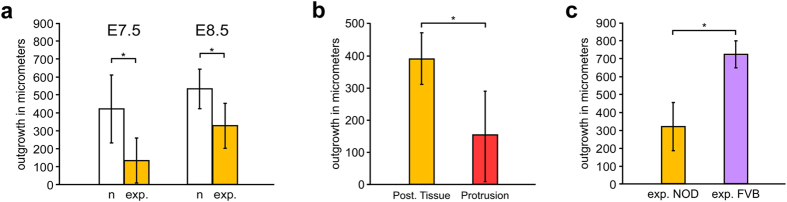
Exposure to maternal diabetes decreases cell migration in explant cultures. Quantification of outgrowth from the explants as the net outward distance migrated by cells at the margin of each explant between hours 6 and 26 after initiation of culture. Asterisks mark statistical significance in a t-test (p < 0.05). (**a**) Posterior tissue explants from diabetes-exposed (exp., orange) NOD embryos display significantly reduced outgrowth compared to explants from normal (n, white) pregnancies at E7.5 (normal n = 10, exposed n = 7; p = 1.7 × 10^−3^; 96.7% power at alpha = 0.05) and E8.5 (normal n = 8, exposed n = 39; p = 6.7 × 10^−4^; 99.7% power at alpha = 0.05). (**b**) Explants of dissected protrusions (red) produced significantly less outgrowth than the corresponding posterior tissue (orange) from the same embryo (n = 12 pairs; p = 6.2 × 10^−5^; 99.9% power at alpha = 0.05) at E8.5. Note the difference in scale of the Y-axis. (**c**) Posterior tissue explants from diabetes-exposed E8.5 NOD embryos (orange; same data as in (**a**); n = 39) exhibit reduced outgrowth compared to explants from diabetes-exposed FVB embryos at E8.5 (purple; n = 6; p = 4.4 × 10^−7^; 100% power at alpha = 0.05).

**Figure 7:**
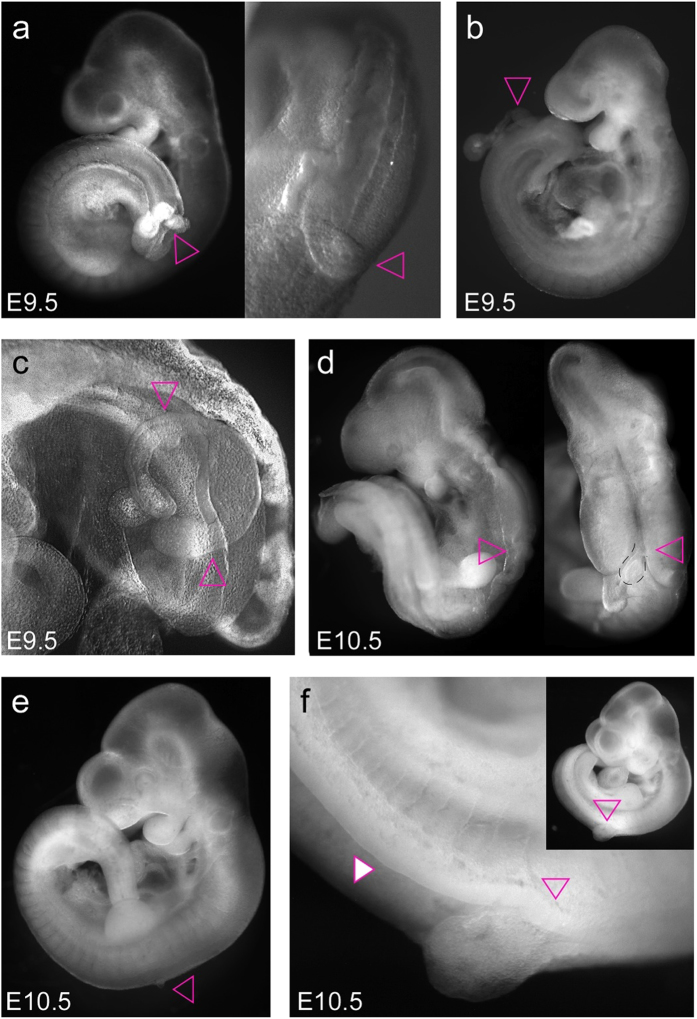
Effect of protrusions on neural tube closure. (**a**) Embryo at 9.5 days of gestation (and close-up) showing a protrusion emerging from underneath the dorsal roof of the neural tube, preventing closure towards the caudal end of the embryo. A schematic drawing and histological analysis are depicted in Supplemental Figure 4. (**b**) Embryo at E9.5 with a long protrusion emerging at approximately hind limb bud level, rendering the neural plate caudal to the protrusion open. (**c**) Embryo at E9.5 featuring a bifurcated protrusion (pink triangles point to the extensions of the protrusion) emanating from the neural tube at the level of the hind limb bud, rendering the neural tube open caudal to the point of emergence. Schematic drawing and histological analysis are provided in Supplemental Figure 4. (**d**) Embryo at E10.5 (lateral and dorsal view) with forebrain and midbrain closed, whereas neurulation failed caudal to the midbrain-hindbrain junction. A small protrusion (stippled black line) can be seen near the hindbrain–spinal cord junction. (**e**) Embryo at E10.5 with very small protrusion at the level of the forelimb bud. Neurulation was completed successfully along the neuraxis except for the small area of the protrusion. (**f**) Embryo at E10.5 with a larger protrusion slightly caudal to the forelimb bud (open triangle). The neural tube is closed rostral to the protrusion, whereas it remains open (white filled triangle) caudally from the protrusion all the way to the end of the neuraxis.
