# Author Correction: Novel mode of defective neural tube closure in the non-obese diabetic (NOD) mouse strain

**DOI:** 10.1038/s41598-022-09478-1

**Published:** 2022-03-30

**Authors:** J. Michael Salbaum, Claudia Kruger, Jacalyn MacGowan, Nils J. Herion, David Burk, Claudia Kappen

**Affiliations:** 1 Pennington Biomedical Research Center, Department of Regulation of Gene Expression, 6400 Perkins Road, Baton Rouge, LA 70808 USA; 2 Pennington Biomedical Research Center Department of Developmental Biology, 6400 Perkins Road, Baton Rouge, LA 70808 USA; 3 Pennington Biomedical Research Center Cell Biology and Bioimaging Core Facility, 6400 Perkins Road, Baton Rouge, LA 70808 USA

Correction to: *Scientific Reports* 10.1038/srep16917, published online 23 November 2015

The Supplementary Information file published with this Article is incomplete, where the supplementary figures and tables are omitted.

The correct Supplementary Information file is now linked to this correction notice.

## Supplementary Information

Supplementary Information.

