# Supplementary material for: Novel Mode of Defective Neural Tube Closure in the Non-Obese Diabetic (NOD) Mouse Strain
Source: Sci Rep. 2015 Nov 23;5:16917. doi: 10.1038/srep16917 (PMC4655353; doi:10.1038/srep16917)
Supplement: Supplementary Information [file srep16917-s2.pdf]

# *Novel Mode of Defective Neural Tube Closure in the Non-Obese Diabetic (NOD) Mouse Strain*

J. Michael Salbaum[1,*], Claudia Kruger[2], Jacalyn MacGowan[1], Nils J. Herion[2], David Burk[3], and Claudia Kappen[2]

SUPPLEMENTAL MATERIAL

**Additional Methods Section**

***Material and Methods.***

*Animal models.*

All animal experiments were performed with prior approval of the Pennington Biomedical Research Center IACUC. Animals were housed with a 12-hour light/dark cycle, with access to food and water ad libitum. Mice of the Non-Obese Diabetic strain (NOD/ShiLtJ, The Jackson Laboratory, Bar Harbor, ME; hence referred to as NOD) as well as male mice of the strain FVB were kept on Purina 5001 diet (calories provided by: Protein, 28.5%; Fat, 13.5%; Carbohydrate, 58%. LabDiet, Purina Mills Inc., Gray Summit, MO); females of the strain FVB were kept on Purina 5015 diet (calories provided by: Protein, 19.8%; Fat, 25.3%; Carbohydrate, 54.8%). Blood glucose levels were monitored weekly in NOD mice; once blood glucose exceeded 250mg/dl, females were considered diabetic, and were used for mating and production of embryos. Diabetic as well as normoglycemic NOD female mice were mated to normoglycemic NOD male mice; once NOD males became hyperglycemic, they were retired from the experiment and no longer used. Chemical induction of diabetes in FVB female mice was done with Streptozotocin (Sigma-Aldrich, St. Louis, MO) according to published protocols[18,71]; once blood glucose levels exceeded 250mg/dl, mice were used for mating with normoglycemic FVB male mice. Noon of the day of the appearance of a

vaginal mating plug was designated day 0.5 of gestation, and embryos were dissected from the uterus at 8.5 days of gestation using a Leica MZ6 stereomicroscope (Leica Microsystems, Buffalo Grove, IL). Extraembryonic endoderm and amnion were removed, and embryos were either fixed in 4% paraformaldehyde in phosphate-buffered saline (PBS) at 4°C, or placed in Tissue-Tek O.C.T. compound (Fisher Scientific, Pittsburgh, PA), frozen, and stored at -80°C.

*Imaging of embryos.*

For imaging, fixed embryos were stained in PBS/4% paraformaldehyde containing DAPI (4',6-diamidino-2-phenylindole) in order to visualize cell nuclei. Embryos were washed in PBS, dehydrated in a graded alcohol series, cleared in BABB (a 1:2 mixture of benzyl alcohol and benzyl benzoate), and imaged on a Leica SP5 Confocal microscope using 2-photon technology. Optical sections were used to generate three-dimensional reconstructions of individual embryos using Imaris software (Bitplane Inc., South Windsor, CT). Computationally generated embryo surfaces as well as virtual sections were used to evaluate the anatomy of dysmorphologies.

*Tissue preparations.*

For laser microdissections, embryos (with 7-9 somite pairs) embedded in O.C.T. were used to produce cryosections at 16μm nominal thickness on a Microm HM560 cryostat. Sections were mounted on glass slides with a PEN (polyethylene naphthalate)

membrane, dried at 50°C for 30 minutes, and stored in a vacuum desiccator. Laser microdissection was performed on a Leica LMD 6000 laser microdissection system. In a set of serial sections from a single embryo, the site of neural tube closure (closure 1) was identified. At the anterior side of the closure, open neural tube tissues was microdissected and collected from the 10 sections preceding the first section showing a closed neural tube. All microdissected tissue segments from an individual embryo were collected into Trizol (Life Technologies, Grand Island, NY) for RNA preparations; RNA was quantified on a Qbit fluorometer (Life Technologies, Grand Island, NY), and stored at -80°C. Ectopic tissue from embryos showing morphogenetic deficiencies of the neural plate was dissected using sterile #55 forceps (Ted Pella Inc., Redding, CA); care was taken not to penetrate the neural plate during removal of the ectopic material. Tissue was directly transferred to Trizol for RNA extraction; RNA was quantified on a Qbit fluorometer, and stored at -80°C.

*Molecular analyses.*

Gene expression profiling was performed by expression tag sequencing (SAGE) on an AB SOLiD 5500XL next-generation sequencing instrument using reagent kits from the manufacturer (Applied Biosystems, Foster City, CA). Sequence reads were aligned to mouse RefSeq transcripts (version mm9) as the reference, utilizing the program SOLiDSAGE (Applied Biosystems). Only uniquely mapped sequence reads were counted to generate the expression count level for each respective RefSeq gene.

Sequencing libraries were constructed from 7ng of laser-microdissected open neural tube material, and yielded an average of 10.2 million mapped reads after quality control. Sequencing libraries from protrusion material were generated from 12ng of total RNA as input material, and yielded an average of 8.9 million mapped reads after alignment and quality control. Differential expression between open neural tube and protrusion tissue was analyzed using the R/Bioconductor program DESeq version 1.81[72]; genes were considered differentially expressed if the adjusted p-value (Benjamini & Hochberg procedure) for the respective comparison was below 0.1. Hierarchical clustering analyses were performed using MeV (Multi Experiment Viewer, http://www.tm4.org/mev/), using Manhattan distance as the correlation metric. Pathway analyses and biological annotation were done using DAVID[73,74]. Validation of sequencing-based expression data was done by qPCR[71,75] using cDNA equivalent to 50pg total RNA per reaction, 4 technical replicates per gene, and normalization to *Polε4* as internal control.

*Histological analyses.*

In situ hybridizations on cryosections using digoxigenin-labelled antisense riboprobes were performed as described[71,76]. For immunohistochemistry[77], cryosections were stained with antibodies against Laminin (Abcam, Cambridge, MA) and Phospho-Histone 3 (directly labeled with Alexa Fluor 488; Biolegend, San Diego, CA); Laminin was detected by indirect immunofluorescence using a goat-anti rabbit Alexa Fluor 594-labeled secondary antibody (Life Technologies, Grand Island, NY). Sections were

counterstained with Hoechst 33342 (Life Technologies, Grand Island, NY), and fluorescence was recorded on an Everest digital microscopy workstation using Slidebook software (Intelligent Imaging Innovations, Inc., Denver, CO).

*Explant cultures.*

Protrusions were microdissected from diabetes-exposed NOD embryos, and posterior tissues containing open neural plate, mesoderm and underlying endoderm were dissected from normal or diabetes-exposed NOD or FVB embryos using glass needles[32] as depicted in Supplemental Figure 3. The explants were placed into 12-well tissue culture plates coated with Matrigel, and cultured in DMEM containing 20% FCS and 2-Mercaptoethanol. Images were taken 6 hours after initiation of the cultures, and again 20 hours later, after a total of 26 hours in culture. At this time point, explants from normal E7.5 NOD embryos are in a phase of active outgrowth (which continues for at least another 3 days without obvious signs of cell death), with the margin of the explant extending outward at an average speed of 0.32 μm/min. (±0.12 μm/min., n=10), as determined from time-lapse videos, using the Leica TIRF DMI6000 system.

These conditions support cell migration and differentiation of mesoderm, including cardiac mesoderm, as evidenced by the presence of rhythmically contractile areas in 7 out of 14 outgrowths from primitive streak explants of E7.5 NOD embryos from normal pregnancies at 26 hours in culture (time-lapse video provided in Supplemental Material). Outgrowth from an explant is expressed as distance migrated by the margin of the explant and was determined as follows: for each image, the size of the area covered by

the explant was determined, and the radius was calculated for a circle of the same area. The radius at the 6h time point of the explant culture was subtracted from the radius for the area covered by the same explant at the 26h time point of culture. Net outgrowth over 20 hours of explants from E8.5 diabetes-exposed NOD embryos was significantly slower ($p=6.7x10^{-4}$) at an average of 0.27 μm/minute (±0.10 μm/min., n=39) than of explants from normal E8.5 NOD embryos at 0.44 μm/minute (±0.09 μm/min., n=8). Outgrowth over 20 hours from explants isolated at E7.5 was also significantly slower (p=0.002) for diabetes-exposed embryos at 0.15 μm/minute (±0.09 μm/min., n=10) than for normal embryos at 0.32 μm/minute (±0.12 μm/min., n=10).

Explants were fixed in 4% paraformaldehyde, washed with PBS, incubated for 20 minutes in TBST, and blocked for one hour in PBS/10% goat serum/1%BSA. For staining, explants were incubated with Alexa488-labeled anti-Vimentin antibody, as well as Alexa594-labeled Phalloidin for 4 hours at room temperature, washed with PBS, incubated with PBS containing DRAQ5, washed again with PBS and coverslipped under Fluoromount G, and fluorescence was recorded on the Everest digital microscopy workstation.

## ***Additional References for Detailed Methods Section***


71 Salbaum, J. M. *et al.* Altered gene expression and spongiotrophoblast differentiation in placenta from a mouse model of diabetes in pregnancy. *Diabetologia* **54**, 1909-1920, doi:10.1007/s00125-011-2132-6 (2011).

72 Anders, S. & Huber, W. Differential expression analysis for sequence count data. *Genome Biol* **11**, R106, doi:10.1186/gb-2010-11-10-r106 (2010).

73 Huang, D. W., Sherman, B. T. & Lempicki, R. A. Systematic and integrative analysis of large gene lists using DAVID bioinformatics resources. *Nat Protoc* **4**, 44-57, doi:10.1038/nprot.2008.211 (2009).

74 Huang, D. W., Sherman, B. T. & Lempicki, R. A. Bioinformatics enrichment tools: paths toward the comprehensive functional analysis of large gene lists. *Nucleic Acids Res* **37**, 1-13, doi:10.1093/nar/gkn923 (2009).

75 Kruger, C. & Kappen, C. Expression of cartilage developmental genes in Hoxc8- and Hoxd4-transgenic mice. *PLoS One* **5**, e8978, doi:10.1371/journal.pone.0008978 (2010).

76 Salbaum, J. M. Punc, a novel mouse gene of the immunoglobulin superfamily, is expressed predominantly in the developing nervous system. *Mech Dev* **71**, 201-204 (1998).

77 Salbaum, J. M., Kruger, C. & Kappen, C. Mutation at the folate receptor 4 locus modulates gene expression profiles in the mouse uterus in response to periconceptional folate supplementation. *Biochim Biophys Acta* **1832**, 1653-1661, doi:10.1016/j.bbadis.2013.04.028 (2013).

**Extended Data/Supplemental Information**

**Legends to Supplementary Figures**

*Supplementary Figure 1*: NOD diabetic pregnancies and folinic acid.

**a**, Supplementation of NOD dams with folinic acid reduced the incidence of neural tube defects in embryos (gestation day 10.5) of diabetic NOD pregnancies from 40.2% (39 NTDs in 97 embryos) to 21% (17 NTDs in 81 embryos). The error bars depict the 95% confidence intervals for each proportion. Fisher's Exact Test revealed that the difference is statistically significant at p=0.006. Maternal hyperglycemia levels at mating and at dissection were not statistically significantly different between supplemented and non-supplemented groups (ANOVA; p=0.51). **b**, Supplementation with folinic acid (12.5mg/kg body weight, once per week by intraperitoneal injection) was initiated at the age of 10 weeks in 56 NOD females, and did not affect the time to subsequent onset of hyperglycemia, when compared to unsupplemented NOD females (n=196). Data are shown in form of a cumulative histogram, and a Kaplan-Meier statistical test revealed no significant difference (p=0.8639) between the two curves (n.s. = not significant).

*Supplementary Figure 2*: Molecular analyses of neurulation-stage FVB embryos.

**a**, Embryo from panel G in Figure 1, where the large protrusion (filled triangle) has been removed from its original position (open triangle) by microdissection, and further used for transcriptome profiling. **b**, Comparative transcriptome profiling between protrusion material and open neural plate. SAGE sequencing revealed expression levels for each gene in the form of tag counts, which were compared with DESeq

software to detect statistically significant expression differences. **c**, Preparation of open neural plate samples by laser microdissection. Embryos at 7-9 somite pairs were prepared; visceral endoderm and amnion were removed, allowing the embryo to relax from the natural U-shaped form to a straight shape. Serial cryosections were prepared and inspected in sequence from the rostral end of the embryo to identify the first section with a closed neural tube. The 10 sections immediately preceding the appearance of the neural tube closure site were subjected to laser microdissection in order to prepare open neural plate material for transcriptome profiling. **d-h**, Specificity of the laser dissection method as demonstrated by analysis *Noto* gene expression, which is typically restricted to node and notochord. **d**, **e** and **f** show qPCR traces obtained from three individual protrusion samples; each was represented by four technical replicates using cDNA equivalent to 50ng of input total RNA. In all of the protrusion samples, four amplification curves were detected, as would be expected for a gene with expression in protrusions. **g** shows qPCR amplification results obtained from four samples of open neural plate, again at four technical replicates each. If *Noto* gene expression were present, one would expect 16 amplification traces. However, only a single trace could be detected, and only at high PCR cycle number, indicating that *Noto* expression is absent from the neural plate samples. **h** depicts *Noto* gene expression as detected by in situ hybridization at E8.5, illustrating the close apposition of the *Noto* expression domain to the closing neural tube. The fact that *Noto* expression was essentially undetectable in open neural plate preparations demonstrated that these preparations were free of contamination with adjacent *Noto*-positive tissue. **i**, results from cluster analysis of gene expression patterns from open neural plate (o1-o4) as well as protrusion samples (p1-

p3) by hierarchical clustering based on Euclidean distance. We sorted genes by smallest adjusted p-value of the comparison between protrusions and open neural plate, and used the top 2000 genes in the analysis. This revealed the close relationship of protrusion samples to each other, yet clearly distinct from open neural plate samples. Likewise, open neural plate samples exhibited close relationship to each other. Yellow color: high expression, blue: low expression. **j**, M/A plot of the comparison between protrusions and open neural plate, revealing expression differences in relation to expression level. Red signifies genes with statistically significantly higher expression in protrusions, whereas genes shown in green have higher expression in open neural plate. Blue dots label differentially expressed genes with known expression in mesoderm; grey color indicates that statistical significance was not reached for the respective gene. Genes where expression was only detected in one side of the paradigm were artificially set at 10 or -10 with respect to the log2 of the expression difference. The plot shows that genes exhibiting differential expression can be detected across 3 orders of magnitude with respect to expression level.

*Supplementary Figure 3*: Explant cultures.

**a**, Schematic of microdissection of tissues for explant cultures from E7.5 embryos. **b**, Potential for outgrowth is higher for explants from developmentally less advanced embryos, as determined by number of somite pairs present. Although the general correlation was weak (low coefficient of determination, $R^2$), the inverse trend was statistically significant in linear regression ($p<0.05$). **c**, The potential for outgrowth from explants, as measured by the distance of extension of the outer margin, is not

dependent on size of the initial explant (weak correlation, not significant: $p>0.05$). **d**, Extent of outgrowth is correlated (high coefficient of determination, $R^2$) to size of the explant at 26 hours, with high statistical significance ($p<0.001$). **e**, Schematic of microdissection of tissues for explant cultures of protrusions and posterior tissue from E8.5 embryos.

*Supplementary Figure 4*: Histological analysis of protrusions.

**a**, Embryo at 9.5 days of gestation (E9.5) featuring a bifurcated protrusion (pink triangles point to each 'arm' of the protrusion) emanating from the neural tube at the level of the hind limb bud. This is the embryo depicted in Figure 7c of the main manuscript. **b**, Schematic representation of a. Green: neural tube and edge of neural plate; blue: open neural plate; red: protrusion. Black lines represent planes of sectioning for immunofluorescence analysis in **c**-**f**. **c**, **d**, **e**, **f**. False-color representation of sections stained with anti-Laminin (red) and Hoechst 33342 (blue). **g**, Embryo at E9.5 (insert) showing a protrusion emerging at the caudal end of the neural tube (this is the embryo depicted in Figure 7a of the main manuscript), with a graphical representation shown in **h**. Immunofluorescence analysis (**i**-**p**) for Laminin (red) and DNA (blue) showed the protrusion emanating from the basal region of the closed neural tube (**j**), then ‘tunneling’ along at variable thickness inside the closed neural tube (**k**, **l**, **m**), merging with the roof of the neural tube (**n**), and finally emanating separately from neural tube tissue (**o**, **p**), leaving open neural plate caudal to the point of emergence.

**Legends to Supplementary Tables**

*Supplementary Table 1*: Protrusion Incidence Data.

Incidence of protrusions in E8.5 embryos from diabetic pregnancies of the NOD strain compared with diabetic pregnancies in the FVB strain after chemical induction of diabetes. Referring to pregnancies, the term ‘affected’ indicates presence of a protrusion in at least one of the embryos of the dam; in case of embryos, the term refers to at least one protrusion in the respective embryo. Blood glucose and litter size values represent the mean plus/minus the standard deviation. Neither maternal blood glucose levels (Anova; NOD: p=0.89; FVB: p=0.14) nor litter sizes (Anova; NOD: p=0.20; FVB: p=0.57) were significantly different between protrusion-affected and non-affected pregnancies.

*Supplementary Table 2*: Genes with Protrusion Prevalence.

The table lists genes with prevalence in protrusions, together with the adjusted p-value from the differential expression test, and the respective expression difference listed as fold-change relative to open neural plate samples. Genes are sorted by increasing adjusted (Benjamini-Hochberg) p-value. Fold differences are given for 'protrusion compared to neural plate'.

*Supplementary Table 3*: Genes with Prevalence in Laser Dissected Open Neural Plate.

The table lists genes with prevalence in open neural plate, together with the adjusted p-value from the differential expression test, and the respective expression difference listed as fold-difference compared to protrusions. Genes are sorted by increasing adjusted (Benjamini-Hochberg) p-value. Fold differences are given for 'protrusion compared to neural plate'.

*Supplementary Table 4*: Genes with protrusion prevalence and previously described expression pattern in node, primitive streak, or mesoderm.

The table lists genes with prevalence in protrusions with the adjusted p-value from the differential expression test, and the respective expression difference, sorted by increasing adjusted (Benjamini-Hochberg) p-value. A search of the MGI database (http://www.informatics.jax.org/) revealed (**i**) a published expression pattern in embryos at Theiler stages 11 to 13, and (**ii**) expression in either node (N), primitive streak (PS), notochord (NC) or mesoderm (M).

*Supplementary Table 5*: Expression of transcription factor targets for T and Cdx in protrusions.

Differential expression analysis revealed *T* and *Cdx2* to be predominant in protrusions when compared to open neural tube. Downstream target genes for these two transcription factors have been described in the literature, and we queried the lists of differentially expressed genes for presence of such transcription factor targets. We detected a total of 57 target genes for *T*: 30 genes originating from a study of *T* targets in ES cells, and 30 from an analysis of *T* targets in chordoma (see references in the

Table); three genes (*Cdh11*, *Epha4*, and *Sema3e*) appear in both studies. Adjusted p-value and fold-difference refer to the protrusion/open-neural-plate comparison; genes are listed by decreasing fold-difference. Interestingly, the ES cell study revealed *Cdx2* as a downstream target of *T*. Targets for *Cdx2* have also been described in ES cells; from that study, we detected 153 genes in the protrusion/open-neural-plate comparison. The data indicate that protrusions express extensive gene regulatory networks driven by key mesodermal transcription factors.
